# Supplementary material for: The Survival of Septic Patients with Compensated Liver Cirrhosis Is Not Inferior to That of Septic Patients without Liver Cirrhosis: A Propensity Score Matching Analysis
Source: J Clin Med. 2022 Mar 15;11(6):1629. doi: 10.3390/jcm11061629 (PMC8951259; doi:10.3390/jcm11061629)
Supplement: Supplementary file 1 [file jcm-11-01629-s001.zip › jcm-1597183-supplementary.pdf]

Table S1. The WLC group versus the LC group before and after propensity score matching.

	Before matching				After matching			
	**WLC n = 712**	**LC n = 64**	**P value**	**SMD[a]**	**WLC n = 114**	LC n = 57	P value	SMD[a]
**Age, years, median (interquartile range)**	69.0 (58.0-79.0)	62.0 (55.3-72.0)	0.003	33.83	65.0 (54.8-76.0)	62.0 (56-74.5)	0.531	9.44
**Male sex, no. (%)**	419 (58.8)	48 (75.0)	0.011	34.78	84 (73.7)	42 (73.7)	1.000	2.32
**APACHE II, median (interquartile range)**	24.0 (19.0-30.0)	25.5 (20.0-32.0)	0.321	16.08	25.5 (21.0-30.0)	25.0 (20.0-31.0)	0.911	10.86
**Comorbidities, no. (%)**								
**Hypertension**	413 (58.0)	19 (29.7)	<0.001	59.23	40 (35.1)	18 (31.6)	0.648	8.92
**Diabetes mellitus**	313 (44.0)	27 (42.2)	0.784	3.37	47 (41.2)	23 (40.4)	0.912	3.80
**Cerebrovascular accident**	141 (19.8)	5 (7.8)	0.019	35.05	7 (6.1)	5 (8.8)	0.525	5.61
**Chronic kidney disease**	221 (31.0)	14 (21.9)	0.126	20.79	28 (24.6)	13 (22.8)	0.800	5.37
**With malignancy**	165 (23.2)	21 (32.8)	0.084	21.59	38 (33.3)	18 (31.6)	0.818	3.54
**Lab data on the day sepsis was confirmed, median (interquartile range)**								
**Hemoglobin, g/dL**	10.8 (9.1-12.5)	9.8 (8.0-12.0)	0.014	35.39	10.1 (8.8-11.8)	10.3 (8.7-12.3)	0.884	1.56
**Platelets, 1000/μL**	195.0 (114.8-271.0)	100.0 (55.0-166.3)	<0.001	79.72	111.0 (65.3-177.0)	107.0 (58.5-174.0)	0.730	3.05

Abbreviations: WLC, without liver cirrhosis; LC, liver cirrhosis; SMD, standardized mean difference; APACHE, Acute Physiology and Chronic Health Evaluation.

a. Imbalance was defined as a standardized difference of > 10%.

Table S2. The WLC group versus the CLC group before and after propensity score matching.

	Before matching				After matching			
	**WLC n = 712**	**CLC n = 24**	**P value**	**SMD[a]**	**WLC n = 88**	CLC n = 22	P value	SMD[a]
**Age, years, median (interquartile range)**	69.0 (58.0-79.0)	61.0 (58.0-72.5)	0.083	27.68	65.0 (55.0-75.0)	59.0 (57.5-69.5)	0.577	0.27
**Male sex, no. (%)**	419 (58.8)	18 (75.0)	0.113	34.58	68 (77.3)	17 (77.3)	1.000	8.17
**APACHE II, median (interquartile range)**	24.0 (19.0-30.0)	26.5 (20.0-32.3)	0.380	23.96	27.0 (22.0-31.0)	26.0 (19.5-30.0)	0.336	1.21
**Comorbidities, no. (%)**								
**Hypertension**	413 (58.0)	9 (37.5)	0.046	41.41	36 (40.9)	9 (40.9)	1.000	2.46
**Diabetes mellitus**	313 (44.0)	10 (41.7)	0.824	4.39	33 (37.5)	9 (40.9)	0.768	5.95
**Cerebrovascular accident**	141 (19.8)	2 (8.3)	0.162	33.06	7 (8.0)	2 (9.1)	0.862	2.94
**Chronic kidney disease**	221 (31.0)	6 (25.0)	0.529	13.32	22 (25.0)	6 (27.3)	0.827	3.09
**With malignancy**	165 (23.2)	8 (33.3)	0.248	22.53	28 (31.8)	7 (31.8)	1.000	4.54
**Lab data on the day sepsis was confirmed, median (interquartile range)**								
**Hemoglobin, g/dL**	10.8 (9.1-12.5)	10.4 (9.3-12.7)	0.850	12.96	10.3 (8.8-11.9)	10.4 (9.3-12.5)	0.598	1.86
**Platelets, 1000/μL**	195.0 (114.7-271.0)	117.0 (45.5-196.0)	0.004	53.19	145.5 (77.5-213.0)	128.0 (45.8-202.3)	0.540	10.41

Abbreviations: WLC, without liver cirrhosis; CLC, Compensated liver cirrhosis; SMD, standardized mean difference; APACHE, Acute Physiology and Chronic Health Evaluation.

a. Imbalance was defied as a standardized difference of > 10%.

Table S3. The WLC group versus the DLC group before and after propensity score matching.

	Before matching				After matching			
	**WLC n = 712**	**DLC n = 40**	**P value**	**SMD[a]**	**WLC n = 99**	DLC n = 33	P value	SMD[a]
**Age, years, median (interquartile range)**	69.0 (58.0-79.0)	62.0 (53.0-72.0)	0.013	37.17	64.0 (54.0-73.0)	64.0 (54.0-75.5)	0.520	4.56
**Male sex, no. (%)**	419 (58.8)	30 (75.0)	0.043	34.71	77 (77.8)	23 (69.7)	0.348	9.56
**APACHE II, median (interquartile range)**	24.0 (19.0-30.0)	25.0 (20.0-32.0)	0.547	10.86	25.0 (21.0-29.0)	25.0 (20.0-30.5)	0.987	4.27
**Comorbidities, no. (%)**								
**Hypertension**	413 (58.0)	10 (25.0)	<0.001	70.59	29 (29.3)	10 (30.3)	0.912	1.32
**Diabetes mellitus**	313 (44.0)	17 (42.5)	0.856	2.74	39 (39.4)	13 (39.4)	1.000	6.32
**Cerebrovascular accident**	141 (19.8)	3 (7.5)	0.054	36.12	7 (7.1)	3 (9.1)	0.704	5.46
**Chronic kidney disease**	221 (31.0)	8 (20.0)	0.140	25.36	21 (21.2)	8 (24.2)	0.716	1.62
**With malignancy**	165 (23.2)	13 (32.5)	0.177	20.87	34 (34.3)	11 (33.3)	0.916	7.69
**Lab data on the day sepsis was confirmed, median (interquartile range)**								
**Hemoglobin, g/dL**	10.8 (9.1-12.5)	9.3 (7.6-11.5)	0.003	47.75	10.1 (8.9-11.8)	9.8 (7.9-11.9)	0.352	19.01
**Platelets, 1000/μL**	195.0 (114.8-271.0)	79.0 (58.3-149.0)	<0.001	99.69	110.0 (61.0-177.0)	104.0 (66.0-157.0)	0.883	9.05

Abbreviations: WLC, without liver cirrhosis; DLC, decompensated liver cirrhosis; SMD, standardized mean difference; APACHE, Acute Physiology and Chronic Health Evaluation.

a. Imbalance was defied as a standardized difference of > 10%.

Table S4: Comparison of qSOFA score, SOFA score, and SOFA sub-scores between the CLC, DLC and WLC groups after matching

	**WLC vs. LC (n = 114 vs. n = 57)**		**WLC vs. DLC (n = 99 vs. n = 33)**		**WLC vs. CLC (n = 88 vs. n = 22)**	
**At initial presentation**	Adjusted OR	**P value[a]**	Adjusted OR	**P value[a]**	Adjusted OR	**P value[a]**
qSOFA score	0.87 (0.62-1.22)	0.420	0.71 (0.30-1.71)	0.449	0.81 (0.48-1.35)	0.416
SOFA score	1.06 (0.97-1.15)	0.183	1.06 (0.95-1.17)	0.310	1.09 (0.95-1.25)	0.209
Respiratory sub-score	0.92 (0.68-1.24)	0.572	0.79 (0.57-1.10)	0.163	1.34 (0.84-2.15)	0.217
Coagulation sub-score	1.03 (0.69-1.53)	0.892	0.87 (0.55-1.37)	0.536	1.47 (0.79-2.75)	0.223
CV sub-score	0.95 (0.71-1.27)	0.714	0.75 (0.51-1.09)	0.134	1.07 (0.70-1.66)	0.747
CNS sub-score	0.99 (0.82-1.20)	0.921	1.01 (0.79-1.28)	0.959	1.08 (0.80-1.46)	0.605
Renal sub-score	1.17 (0.91-1.50)	0.218	1.43 (0.99-2.01)	0.060	1.06 (0.71-1.58)	0.775
**Day 1**						
qSOFA score	0.90 (0.58-1.37)	0.614	0.63 (0.36-1.12)	1.117	0.83 (0.45-1.55)	0.562
SOFA score	1.06 (0.97-1.15)	0.194	1.09 (0.98-1.22)	0.118	1.10 (0.95-1.26)	0.210
Respiratory sub-score	1.05 (0.82-1.35)	0.705	0.93 (0.69-1.26)	0.636	1.21 (0.81-1.82)	0.352
Coagulation sub-score	1.29 (0.85-1.96)	0.228	1.34 (0.85-2.11)	0.210	1.35 (0.79-2.32)	0.273
CV sub-score	0.80 (0.65-0.98)	0.028	0.71 (0.53-0.95)	0.019	0.85 (0.65-1.12)	0.253
CNS sub-score	1.15 (0.88-1.50)	0.307	1.16 (0.85-1.59)	0.337	1.55 (0.96-2.53)	0.076
Renal sub-score	1.11 (0.88-1.40)	0.363	1.35 (0.97-1.87)	0.072	0.92 (0.64-1.34)	0.678
**Day 3**						
qSOFA score	0.96 (0.59-1.56)	0.868	1.04 (0.56-1.94)	0.895	0.76 (0.36-1.60)	0.466
SOFA score	1.05 (0.97-1.14)	0.264	1.17 (1.03-1.32)	0.017	0.98 (0.85-1.12)	0.714
Respiratory sub-score	1.02 (0.75-1.39)	0.916	0.95 (0.65-1.38)	0.775	1.04 (0.63-1.71)	0.888
Coagulation sub-score	1.26 (0.86-1.84)	0.238	1.83 (1.08-3.10)	0.024	1.13 (0.69-1.84)	0.639
Liver sub-score	1.87 (1.25-2.80)	0.002	2.23 (1.38-3.61)	0.001	1.21 (0.70-2.09)	0.487
CV sub-score	0.89 (0.69-1.12)	0.336	0.88 (0.61-1.25)	0.465	0.90 (0.61-1.32)	0.577
CNS sub-score	0.98 (0.73-1.31)	0.882	1.04 (0.73-1.47)	0.837	1.05 (0.68-1.61)	0.829
Renal sub-score	1.15 (0.92-1.44)	0.227	1.22 (0.90-1.67)	0.201	0.99 (0.69-1.44)	0.970
**Day 7**						
qSOFA score	1.02 (0.64-1.63)	0.937	1.32 (0.76-2.30)	0.329	1.00 (0.42-2.35)	0.994
SOFA score	1.03 (0.95-1.12)	0.447	1.21 (1.06-1.39)	0.004	0.92 (0.78-1.08)	0.319

Respiratory sub-score	1.03 (0.71-1.50)	0.874	1.11 (0.73-1.69)	0.633	1.88 (0.86-4.09)	0.113
Coagulation sub-score	1.13 (0.81-1.57)	0.471	2.84 (1.56-5.21)	0.001	0.82 (0.45-1.49)	0.514
CV sub-score	1.03 (0.76-1.40)	0.853	1.23 (0.87-1.74)	0.234	0.47 (0.15-1.55)	0.218
CNS sub-score	0.80 (0.58-1.10)	0.170	1.00 (0.69-1.45)	0.987	0.71 (0.44-1.16)	0.174
Renal sub-score	1.20 (0.93-1.54)	0.160	1.43 (1.00-2.03)	0.048	0.98 (0.661.46)	0.931

Abbreviations: qSOFA, Quick SOFA; SOFA, Sequential Organ Failure Assessment; WLC, without liver cirrhosis; LC, liver cirrhosis; DLC, decompensated liver cirrhosis; CLC, compensated liver cirrhosis; OR, odds ratio; CV, cardiovascular; CNS, central nervous system.

a. Conditional logistic regression
